# Supplementary material for: Low-Temperature Storage Improves the Over-Time Stability of Implantable Glucose and Lactate Biosensors
Source: Sensors (Basel). 2019 Jan 21;19(2):422. doi: 10.3390/s19020422 (PMC6359396; doi:10.3390/s19020422)
Supplement: Supplementary file 1 [file sensors-19-00422-s001.zip › sensors-422128-SI.pdf]

Low-Temperature Storage Improves the Over-Time Stability of Implantable Glucose and Lactate Biosensors

Giulia Puggioni †, Giammario Calia †, Paola Arrigo, Andrea Bacciu, Gianfranco Bazzu, Rossana Migheli, Silvia Fancello, Pier Andrea Serra * and Gaia Rocchitta

[1] Medical, Surgical and Experimental Sciences, University of Sassari, Viale San Pietro 43/b, 07100 Sassari, Italy; giuliamariagrazia@gmail.com (G.P.); gmcalia@uniss.it (G.C.); pa1989@live.it (P.A.); andreabacciu90@gmail.com (A.B.); gbazzu@uniss.it (G.B.); rmigheli@uniss.it (R.M.); sfancello@uniss.it (S.F.); grocchitta@uniss.it (G.R.)

* Correspondence: paserra@uniss.it.

† The authors share first name.

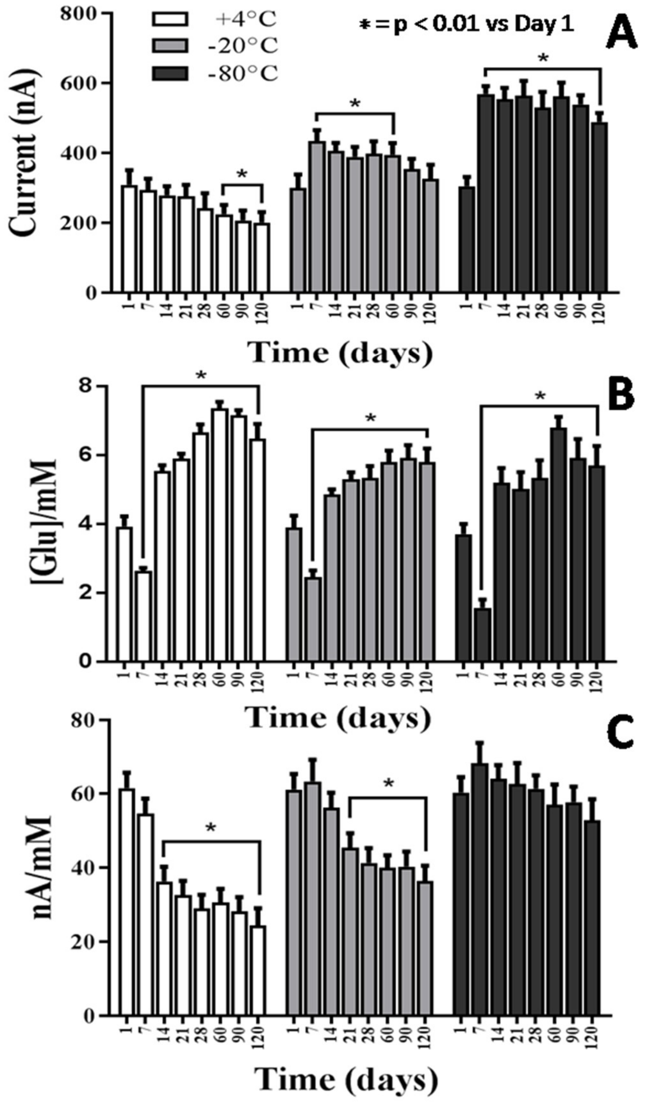

Figure S1. Bar chart describing the variations of V_{MAX} (Panel A, white bars), K_M (Panel B, light gray bars) and LRS (Panel C, dark gray bars) of GB1 design Pt_c/PPD/[PEI(0.5%)-GOx]$_5$/PU(5%) in the defined range of 120 days. Values are expressed as mean ± SEM. *p<0.01 vs Day 1.

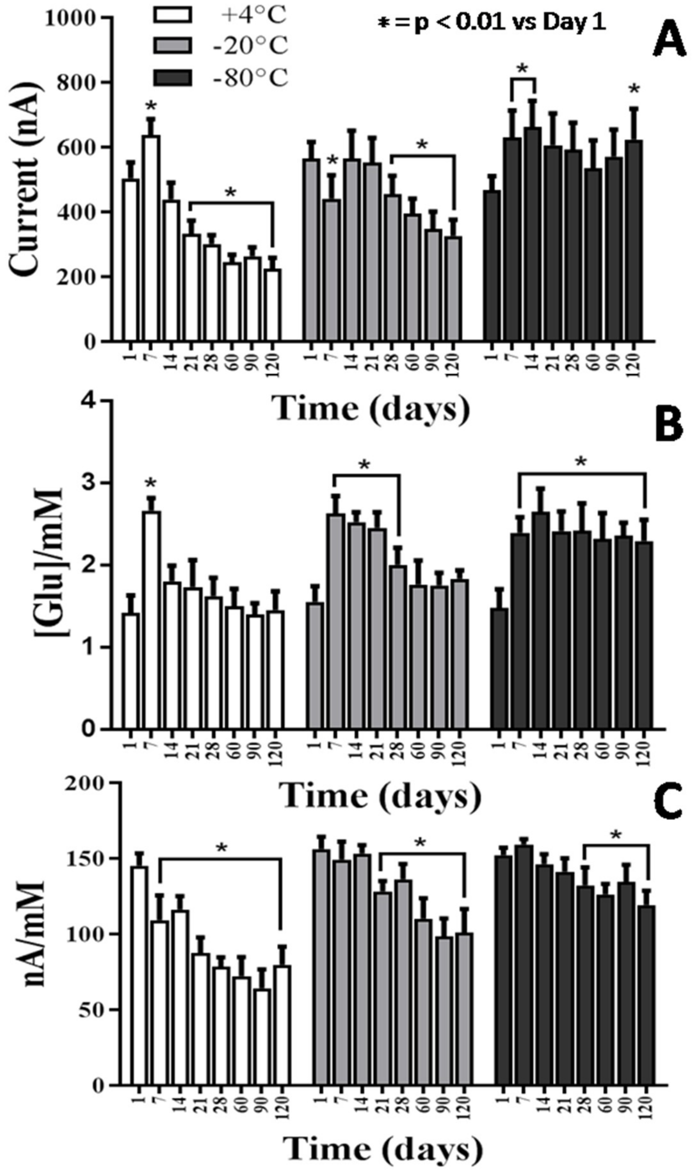

Figure S2. Bar chart describing the variations of V_{MAX} (Panel A, white bars), K_M (Panel B, light gray bars) and LRS (Panel C, dark gray bars) of GB2 design Pt_c/PPD/[PEI(0.5%)-GOx]$_5$/GTA(1%)-BSA (2%) in the defined range of 120 days. Values are expressed as mean ± SEM. *p<0.01 vs Day 1.

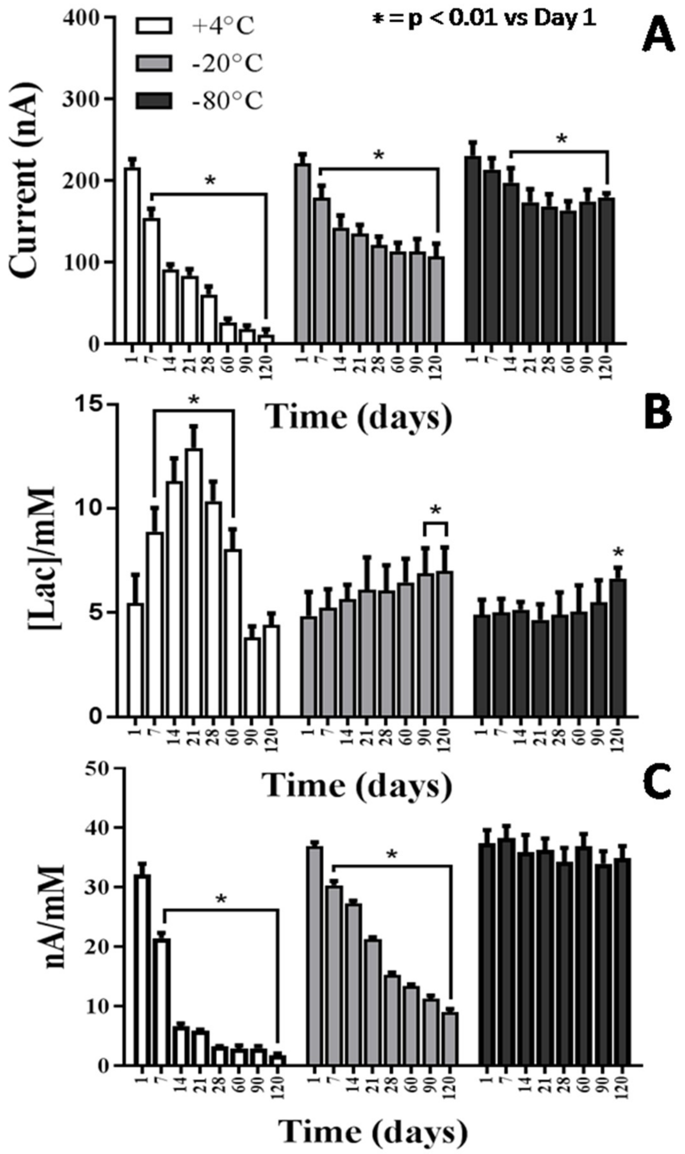

Figure S3. Bar chart describing the variations of V_{MAX} (Panel A, white bars), K_M (Panel B, light gray bars) and LRS (Panel C, dark gray bars) of LB1 design Pt_c/PPD/[PEI(0.5%)-LOx]$_5$/GTA(1%)-BSA (2%) in the defined range of 120 days. Values are expressed as mean ± SEM. *$p<0.01$ vs Day 1.

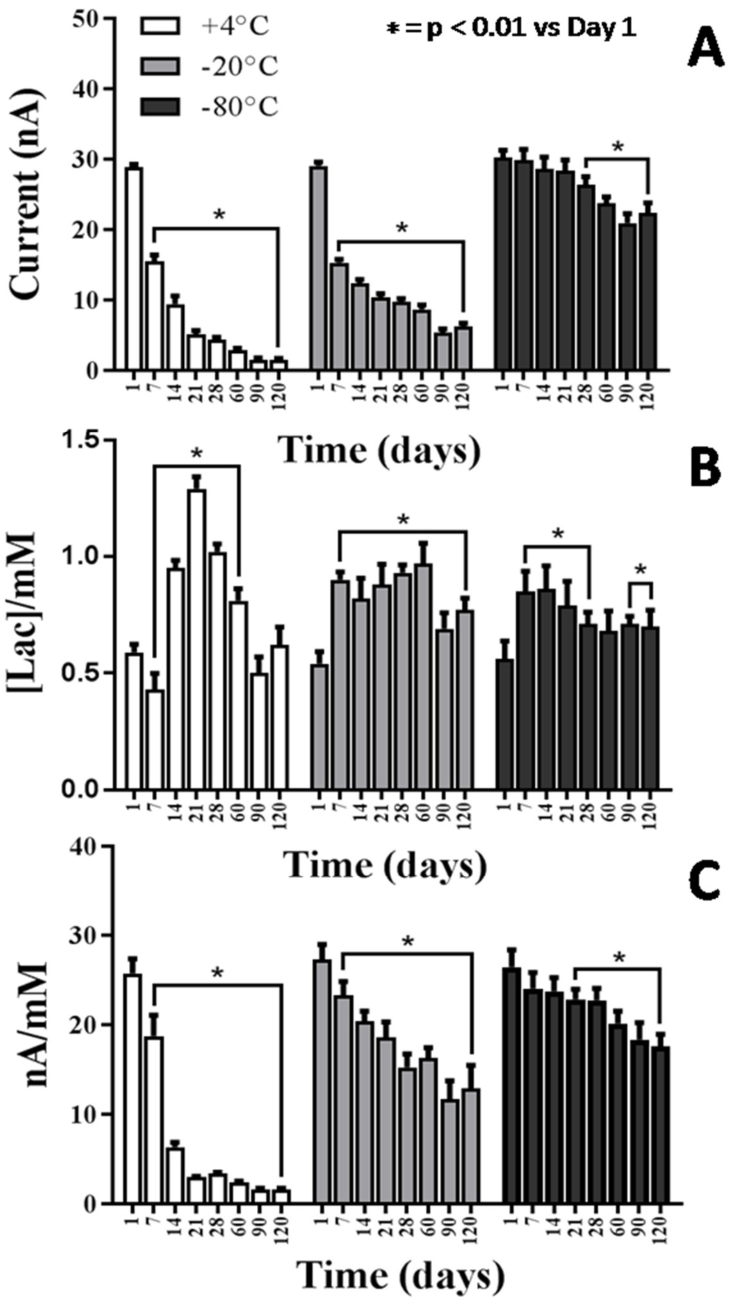

Figure S4. Bar chart describing the variations of V_{MAX} (Panel A, white bars), K_M (Panel B, light gray bars) and LRS (Panel C, dark gray bars) of LB2 design Pt_c/PPD/[PEI(0.5%)-LOx]$_5$/GTA(1%)-BSA (2%) in the defined range of 120 days. Values are expressed as mean ± SEM. *p<0.01 vs Day 1.
